# Suitability of SBR for Wastewater Treatment and Reuse: Pilot-Scale Reactor Operated in Different Anoxic Conditions

**DOI:** 10.3390/ijerph17051617

**Published:** 2020-03-02

**Authors:** Omar Alagha, Ahmed Allazem, Alaadin A. Bukhari, Ismail Anil, Nuhu Dalhat Mu'azu

**Affiliations:** 1 Environmental Engineering Department, College of Engineering A13, Imam Abdulrahman Bin Faisal University, Main Campus, Dammam 31451, Saudi Arabia; 2 Civil and Environmental Engineering Department, College of Engineering, King Fahad University of Petroleum and Minerals, Dhahran 31261, Saudi Arabia; 3 The Electricity & Co-Generation Regulatory Authority, Riyadh 12711, Saudi Arabia

**Keywords:** sequencing batch reactor, pilot-scale treatment, nitrogen and phosphorus removal, domestic wastewater treatment, sustainable wastewater management, arid regions

## Abstract

The present study investigates the performance of a pilot-scale Sequencing Batch Reactor (SBR) process for the treatment of wastewater quality parameters, including turbidity, total suspended solids (TSS), total solids (TS), nitrogen (ammonia (NH_3_–N), nitrite (NO_2_^−^), and nitrate (NO_3_^−^), phosphate (PO_4_^3−^), the chemical oxygen demand (COD), and the 5-day biological oxygen demand (BOD_5_), from municipal wastewater. Two scenarios, namely, pre-anoxic denitrification and post-anoxic denitrification, were investigated to examine the performance of a pilot-scale SBR on the wastewater quality parameters, particularly the nitrogen removal. The correlation statistic was applied to explain the effects of operational parameters on the performance of the SBR system. The results revealed that the post-anoxic denitrification scenario was more efficient for higher qualify effluent than the first scenario. The effluent concentrations of the targeted wastewater quality parameters obtained for the proposed SBR system were below those of the local standards, while its performance was better than that of the North Sewage Treatment Plant, Dharan, Eastern province, Kingdom of Saudi Arabia (KSA), in terms of the BOD_5_, COD, TN, and PO_4_^3-^ treatment efficiencies. These results indicated the suitability of SBR technology for wastewater treatment in remote areas in the KSA, with a high potential of reusability for sustainable wastewater management.

## 1. Introduction

The activated sludge system (ASS) has been the conventional technique adopted by the majority of municipal wastewater treatment plants, globally, for wastewater treatment [1]. In recent years, the sequencing batch reactor (SBR), an enhanced form of the ASS process, has become a popular replacement technique due to its unique merits [2]. The SBR is one of the integrated systems for anaerobic-aerobic bioreactors in which the wastewater is treated in a fill and draw method [3]. The process of a typical SBR mainly consists of five steps, conducted in the following sequence: filling, reacting, settling, decanting, and idle [4,5]. In the first phase (the filling phase), the wastewater influent and additional enhancement substrate (if required) are added, from which up to 75% of the container reactor volume can be occupied. During the filling period, mixing with or without aeration can be practiced. During the reaction phase, which is the second step of the SBR process, considering specific environmental conditions, the substrate (ammonium nitrogen) is oxidized to nitrates, and the change of its form takes place in the reactor, which contains particular bacteria responsible for the substrate degradation [6]. In the third phase, the settling period involves the separation of the solids from the effluent, providing the colloidal solids and the suspended solids with enough time to accumulate and settle [7]. The decanting step is the fourth stage of the process, in which the removal of treated effluent from the treatment tank is achieved. In the last step (the idle phase), the sludge is removed from the container, and this phase is only necessary for multi-chamber systems [8,9,10].

The use of the SBR process is more prevalent in industrial wastewater treatment because of its compactness and the high efficiency of the chemical oxygen demand (COD), biological oxygen demand (BOD), and ammonia nitrogen removal [11,12,13]. However, SBR process deployment for domestic wastewater treatment is rare, since domestic wastewater usually needs large-capacity plants due to higher wastewater flow rates [14]. Conventional biological wastewater treatment plants using ASS have some disadvantages, though they can work adequately under proper installation and maintenance [15]. However, the SBR process, as an enhanced form of the conventional treatment system, presents flexibility for the treatment of variant influents, the lowest operator interaction, an alternative for aerobic and anaerobic environments in the same chamber, an excellent oxygen interaction with microorganisms and substrates, a lesser footprint, a superior removal efficiency, and the requirement of less energy input [16,17,18]. These benefits validate the increased interest in the adoption of the SBR process for the treatment of both municipal and industrial wastewater [19]. Nevertheless, anaerobic and aerobic cycle times in the SBR system may generate some issues regarding controlling the anaerobic-aerobic microbial groups and, therefore, selection and enhancement of the biomass become necessary [20]. Regulation of the anoxic and oxic phases during the SBR process can enrich the targeted microbial population, and hence, improve the process efficacy. The duration of the phases, dissolved oxygen concentration, and mixing conditions can be changed in accordance with the particular requirements of the treatment plants [20].

There are two main types of biological denitrification: pre-anoxic and post-anoxic processes [11,21,22]. In pre-anoxic denitrification, the anoxic phase is located upstream of the aerobic phase. The electron donor in the anoxic container is the available organic substrate. In the post-anoxic operation, the oxic phase followed by the anoxic basin is the primary process. The endogenous decay of biomass provides the source of electron donor in the anoxic tank [23]. The organic substrate in the raw wastewater consumed in the process of carbon removal and nitrification simultaneously occurs [24].

In biological nitrogen removal, the scarcity of biodegradable organic substrate to nitrogen compounds (i.e., low C/N ratio) is considered one of the restrictive factors [25]. Along with other heterotrophs, denitrifying bacteria are known to compete for a carbon source. Only a small carbon to nitrogen ratio in the influent accelerates the carbon deficiency, producing unstable instantaneous denitrification. The average COD/TKN ratio in domestic wastewater was reported to be beneath 6 [26]. Beccari, et al. [27] validated that the biological removal of nitrogen can be appropriately accomplished with a COD/TKN ratio of 13. Fontenot, et al. [28] proved that the C/N ratio of 10:1 provided excellent results in terms of the maximum nitrogen and carbon removal from wastewater. In 2012, Jin and Li [29] investigated the capability of a laboratory-scale SBR in the removal of nitrogen and phosphorus. Using different C/N ratios, they found an optimum C/N ratio (10:1) for TN removal. Guo, et al. [30] reported a study that investigated the effect of sludge fermentation on the nitrogen removal efficiency from low C/N wastewater using a 9 L SBR. By controlling the pH and dissolve oxygen (DO), they found that the removal efficiency of TN reached up to 93.5%.

As mentioned earlier, pre-anoxic denitrification consists of an anoxic zone followed by an oxic zone; in the SBR system, the pre-anoxic denitrification can be provided by increasing the filling time without any aeration (anoxic zone) and decreasing the settling time. For the post-anoxic denitrification in which the oxic zone is applied before the anoxic zone, the settling time is increased (without any aeration) and the filling time is decreased (regardless of whether the aeration step takes place or not).

The purpose of this study was to investigate the performance of a pilot-scale SBR for the treatment of municipal wastewater by testing different anoxic conditions in the SBR reactor and comparing the effluent quality with those of conventional ASS. Therefore, the study experimentally examined the suitability of the SBR process as a decentralized wastewater treatment system for evaluating its effectiveness as an alternative system for sustainable wastewater treatment and management in remote arid areas of the Kingdom of Saudi Arabia (KSA), where conventional treatment processes are lacking.

## 2. Materials and Methods

### 2.1. Characteristics of the Studied Wastewater

The raw wastewater (influent) and treated wastewater (effluent) samples were collected on a daily basis from the North Sewage Treatment Plant (NSTP), Dhahran, KSA. The NSTP is an activated sludge wastewater treatment plant with an average flow rate of 52,000 m^3^ d^−1^, receiving only domestic wastewater from Dhahran and environs. The collected samples were immediately transferred to the Biological Processes Laboratory of Environmental Engineering Department, Imam Abdulrahman Bin Faisal University, and analyzed for the pH, turbidity (turb.), total suspended solids (TSS), total solids (TS), nitrite-nitrogen (NO_2_–N), nitrate-nitrogen (NO_3_–N), ammonia-nitrogen (NH_3_–N), total nitrogen (TN), phosphate (PO_4_^3−^), chemical oxygen demand (COD), and 5-day biological oxygen demand (BOD_5_), according to the procedures described in “The Standard Methods for the Examination of Water and Wastewater” [31]. Table 1 indicates the descriptive statistical analysis summary of the parameters measured for the influent of NSTP during the studied period.

### 2.2. Pilot-Scale SBR System Description

**Figure 1:**
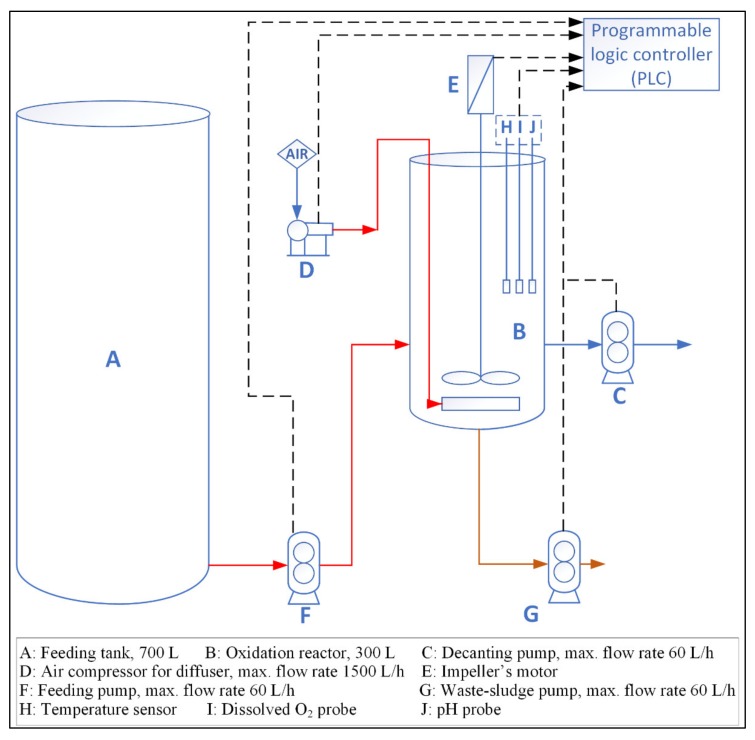
Schematic diagram of the pilot-scale sequencing batch reactor (SBR).

In this study, pre-denitrification and post-denitrification processes were investigated in the SBR process to find the most appropriate method for the removal of pollutants. Figure 1 shows a schematic diagram of the pilot-scale SBR system used in the present work. The system consists of a feed tank with a capacity of 700 L and a cylindrical oxidation reactor with a capacity of 300 L, which were made from inert, transparent methacrylate material. The oxidation reactor includes a stainless-steel agitator and air diffuser. The system comprised (1) feeding, decanting, and waste-sludge pumps with a maximum flow-rate of 60 L h^−1^; (2) a diaphragm compressor with a stainless-steel body and flow rate of 1.2 Nm^3^ h^−^^1^; and (3) a flowmeter for measuring the feed flow rate of air to the reactor with a range of 0–1500 NL h^−^^1^. The pilot-SBR reactor system has a board-type microprocessor-controlled pH-meter, temperature sensor, and DO-meter with a detection range between 0 and 10 ppm.

### 2.3. Experimental Setup and Operation of the SBR System

The NSTP wastewater influent samples were obtained on a daily basis from the inlet point to the aeration tank, where the influent is screened through coarse and fine screens to remove any large or fine particles. The samples were collected in 250 L containers and transferred within 30 min to the SBR reactor, ensuring that the development of the anaerobic condition was avoided prior to the SBR treatment process. No unusual foam or grease buildup was observed for the collected samples.

Two scenarios were applied to investigate the treatment efficiency of the SBR system. In the pre-anoxic denitrification, anoxic and oxic zones were employed sequentially. The pre-anoxic denitrification in the SBR was controlled by increasing the filling time and decreasing the settling time without any aeration (anoxic zone). In the post-anoxic denitrification, the cycle of oxic and-anoxic zones was established. In this cycle, the settling time was increased, and the filling time was decreased (without any aeration). Each cycle consisted of three runs, with different batch numbers and various combinations of filling time (T_f_), aeration time (T_a_), settling time (T_s_), and decanting time (T_d_). Table 2 shows the T_f_, T_a_, T_s_, and T_d_ values that were applied to each batch during pre-anoxic denitrification and post-anoxic denitrification processes.

The operating volume of the oxidation reactor was kept at 240 L. The startup was initiated by filling the oxidation reactor with the aerated wastewater collected from the aeration tank of NSTP. The preliminary cycle was obtained within 8 h. The second preliminary batch was started by adding raw sewage from the aeration tank intake; as such, there was no need for adding sludge. The concentrations of mixed liquor suspended solids (MLSS) and mixed liquor volatile suspended solids (MLVSS) were kept at around 3500 and 1800 mg L^−1^, respectively. The average sludge retention time (SRT) was calculated to be 15 days, which is in good agreement with the reported values for the SBR process treatment of domestic wastewater [32,33]. The DO concentration during the aeration phase was measured by using an on-line DO probe and controlled simultaneously via an automated air compressor to maintain the DO concentration near 2 mg L^−1^. No external source of carbon was added to the SBR process since the average C/N ratio of the influent was 9. Gentle agitation was ensured by using an axial flow 3-blade impeller to retain the liquor in a well-mixed condition in filling and aeration phases. The pilot SBR system was operated in a temperature-controlled laboratory, which resulted in an average reactor temperature of 26.8 ± 0.8 °C. All operations throughout the SBR experiments were controlled by a programmable logic controller (PLC) integrated into the system.

The removal efficiency (RE, %) of each wastewater quality parameter was calculated by using Equation (1):

(1) $$\mathrm{RE}\left(\%\right)=\frac{C_{\mathrm{Inf}}-C_{\mathrm{Eff}}}{C_{\mathrm{Inf}}}\times 100,$$

where C_Inf_ is the concentration of the parameter in the influent, and C_Eff_ is the concentration of the parameter in the effluent. Each analysis for the quantitative determination of wastewater quality parameters was performed in triplicate, and average results were reported. The statistical analyses of the dataset and the correlation statistics were obtained with the aid of SPSS (Statistical Package for the Social Sciences, IBM Corp., Armonk, NY, USA) Released 2016. IBM SPSS Statistics for Windows, Version 24.0. (IBM Corp, Armonk, NY, USA).

## 3. Results and Discussion

### 3.1. Nitrogen Removal

**Figure 2:**
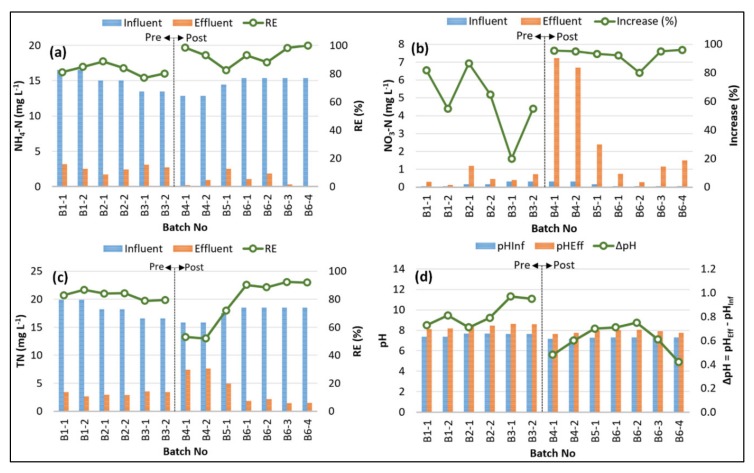
Variations of (**a**) nitrogen (NH_3_–N), (**b**) nitrate-nitrogen (NO_3_–N), (**c**) total nitrogen (TN), and (**d**) pH.

The variations of influent and effluent concentrations and removal efficiencies (RE) of NH_3_–N, NO_3_–N, and TN during the SBR pilot-scale batch experiments are shown in Figure 2. The average effluent concentrations of NH_3_–N after pre-anoxic denitrification scenario I (SCI) and oxic post nitrification scenario II (SCII) were found to be 2.59 ± 0.48 mg L^−1^ and 0.98 ± 0.86 mg L^−1^, respectively. The highest NH_3_–N removal efficiency of 99.9% was achieved by SCII (Batch# 6–4), while SCI resulted in the lowest NH_3_–N removal efficiency of 77.0% (Batch# 3–1). These results reveal that more favorable conditions for the oxidation of NH_3_–N were provided by SCII, where the average T_a_/T_total_ = 0.36, while the average T_a_/T_total_ in SCI was 0.24.

The primary product of nitrification during the SBR experiments was NO_3_–N, which accumulated up to 7.2 mg L^−1^, while the NO_2_–N concentration was always below the quantification limit. The average NO_3_–N accumulation ratios (% increase) in SCI and SCII were calculated as 60.6% (±21.9) and 92.5% (±5.19), respectively. The level of NO_3_–N accumulation could be ascribed to (1) the higher activity of ammonia-oxidizing bacteria (AOB) and more effective nitrification in SCII, and (2) the more efficient denitrification process and nitrite-oxidizing bacteria (NOB) activity in SCI, where the ratio of total anoxic period to total time ((T_f_ + T_s_)/T_total_) was higher than that of SCII. High NO_3_–N accumulation has been reported by several previously published SBR studies [8,21,33,34].

The TN removal efficiency is dependent on the performances of nitrification and denitrification processes [22,35,36]. The average TN concentrations measured for effluent samples of SCI and SCII were 3.13 ± 0.33 mg L^−1^ and 3.83 ± 2.57 mg L^−1^, respectively. The average TN removal efficiencies of SCI and SCII were 82.7% (±2.78) and 77.1% (±16.9), respectively. The highest TN removal efficiency reached 92% after the SBR cycles in Batch# 6–3 and 6–4 were completed. It is evident from Figure 2a–c that the nitrification performance played a predominant role compared to the denitrification process in reaching the highest TN removal in these two cases. These findings showed that post-anoxic denitrification (SCII) could perform better TN removal than pre-anoxic denitrification (SCI), corroborating earlier reported studies [21,37].

The pH of the SBR experiments conducted in this work was not controlled, and the effluent pH values ranged between 7.6 and 8.6, with an average of 8.1. The pH change (Δ_pH_ = pH_Eff_ − pH_Inf_) throughout the SBR experiments is indicated in Figure 2d. It can be concluded from Figure 2d that pH_Eff_ was always greater than pH_Inf_, which could be attributed to the domination of the denitrification process in SBR experiments, inducing an increase in pH. The average Δ_pH_ values calculated for SCI and SCII were 0.83 ± 0.10 and 0.61 ± 0.11, respectively. High NH_3_–N removal efficiencies and high NO_3_–N accumulation rates observed in SCII were accompanied by low Δ_pH_ values, implying that nitrification was the governing process and repressed the excessive pH increase due to the denitrification process since the allocated time for aeration was greater than that of anoxic periods in SCII (T_a_ > T_f_ + T_s_). On the other hand, SCI having a longer period of time assigned for anoxic conditions (T_f_ + T_s_ > T_a_) yielded greater Δ_pH_ because of the effective denitrification that increased the alkalinity of the solution [30,38,39,40].

### 3.2. COD, BOD_5_, and PO_4_^3−^ Removal

**Figure 3:**
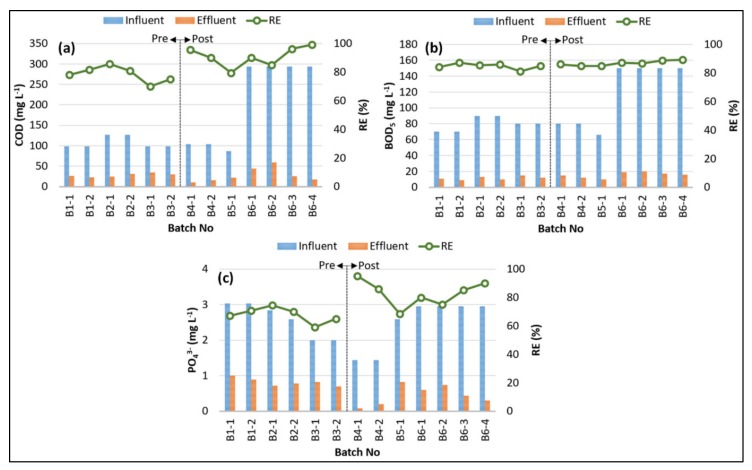
Variations and removal efficiencies of (**a**) the chemical oxygen demand (COD), (**b**) the 5-day biological oxygen demand (BOD_5_), and (**c**) phosphate (PO_4_^3−^).

Figure 3 depicts the changes in influent and effluent concentrations and removal efficiencies (RE) of COD, BOD_5_, and PO_4_^3−^ through the SBR plot scale batch experiments. The average effluent COD concentrations of SCI and SCII were computed to be 28.0 ± 3.84 and 27.7 ± 16.2 mg L^−1^, respectively. Even though the average effluent COD concentrations of both scenarios were very close, the average COD removal efficiency of the SCII was greater than that of SCI due to the higher average influent COD concentration of SCII. The average COD removal efficiencies of SCI and SCII were 78.6% (±5.0) and 90.7% (±6.4), respectively. The SCII attained the highest COD removal efficiency of 99.1% at the end of Batch# 6–4, which could be clarified by the faster filling phase, with the longer aeration and settling periods dedicated in SCII eventuating a better COD removal efficiency [20]. As a result, it implies that the post-anoxic denitrification scenario was more effective than the pre-anoxic denitrification scenario in terms of COD removal.

BOD_5_ removal could be used as an indicator of the treatment efficiencies of the biological treatment processes [41,42]. The influent BOD_5_ concentrations indicated important decreases after the SBR experiments, with average removal efficiencies of 84.9% (±1.84) and 86.8% (±1.60) for SCI and SCII, respectively. As demonstrated in Figure 3b, the BOD_5_ removal efficiency did not fluctuate much and resulted in a low standard deviation value (±1.98) for all the experiments. The highest BOD_5_ removal efficiency of 88% was observed for Batch# 6, where the influent BOD_5_ concentration was the maximum among all the SBR experiments. The SCII provided higher removal efficiencies for both COD and BOD_5_ parameters, even for their higher influent concentrations, compared to the SCI, which might be ascribed to the more effective oxidation of organic matter allowed by batch experiments in SCI.

The average influent PO_4_^3−^ concentration decreased from 2.52 ± 0.57 to 0.62 ± 0.27 mg L^−1^, with an average removal efficiency of 75.9% (±10.3), including both scenarios. The PO_4_^3−^ removal efficiency of both SCI and SCII indicates similar values in terms of COD and NH_3_–N removal efficiencies. The SCII provided a better PO_4_^3−^ removal rate (82.8%) than that of SCI (67.7%), which was also experienced for NH_3_–N, COD, and BOD_5_ removals. In the case of simultaneous nitrogen and phosphorus treatment, the competition between NOBs and phosphorus-accumulating organisms (PAOs) leads to unsteady PO_4_^3−^ removal, unless the COD amount of influent is sufficient [37,43,44]. In this work, the COD content of the influent was not a critical limiting factor for PO_4_^3−^ removal as it was most likely provided by the available carbon throughout the oxic period, particularly in SCII. In addition to this, Kundu, Debsarkar and Mukherjee [39] addressed that higher phosphorus uptakes could be achieved when SRT was less than 25 days, which could also support the efficient PO_4_^3−^ uptake results obtained here since the SRT of SBR experiments was 15 days.

### 3.3. Turbidity, TSS, and TS Removal

**Figure 4:**
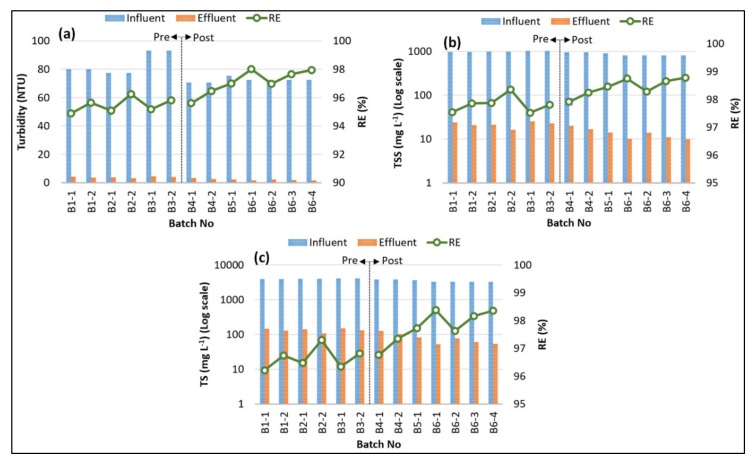
Variations and removal efficiencies of (**a**) turbidity, (**b**) total suspended solids (TSS), and (**c**) total solids (TS).

The variations of influent and effluent concentrations and removal efficiencies (RE) of Turbidity, TSS, and TS are indicated in Figure 4. The average influent values of turbidity, TSS, and TS were 77.5 ± 7.36 NTU, 926 ± 83.3 mg L^−1^, and 3705 ± 333 mg L^−^^1^, respectively. These values were reduced for the effluent to 2.88 ± 0.99 NTU, 17.4 ± 5.14 mg L^−^^1^, and 104 ± 34.4 mg L^−^^1^, with average removal efficiencies of 96.3%, 98.2%, and 97.2%, respectively. The removal rates of turbidity, TSS, and TS exhibited similar patterns, and the Pearson correlation coefficients (*p*) between Turbidity_RE_ and TSS_RE_, Turbidity_RE_ and TS_RE_, and TSS_RE_ and TS_RE_ were computed to be 0.96, 0.99, and 0.98, respectively (within a 95% confidence interval). These very strong correlations calculated for the removal efficiencies of turbidity, TSS, and TS parameters suggest that their removals were controlled by a common mechanism, which is the sedimentation phase of the SBR experiments. The SCII achieved slightly higher removal efficiencies for all these parameters in comparison with the SCI. This finding could be linked to the longer settling times given in the SCII compared to the SCI, providing the colloidal solids and the suspended solids with enough time to accumulate and settle [14,42].

### 3.4. Effects of Parameters on the SBR System Performance

The correlation statistics were applied to removal efficiencies of wastewater quality parameters, the NO_3_–N accumulation rate (NO_3_–N_AR_), and operational parameters of the SBR experiments in order to statistically explain the effects of operational parameters on the performance of the SBR system. The correlation matrix, including *p* values between each parameter, is shown in Table 3. As observed in Section 3.3., Turb._RE_, TSS_RE_, and TS_RE_ were strongly correlated with each other, and they indicated very strong correlations with the ratio of the settling period to the total batch runtime (T_s_/T_total_). Increasing T_s_/T_total_ provides more time for particles to be settled down efficiently, which importantly increases Turb._RE_, TSS_RE_, and TS_RE_.

The ratio of the oxic period to total batch runtime (T_a_/T_total_) was assessed in relation to its impact on the performance parameters of the SBR system. The T_a_/T_total_ ratio presented moderate-to-very strong positive correlations with BOD_5RE_ (0.51), TN_RE_ (0.61), NO_3_-N_AR_ (0.74), NH_3_-N_RE_ (0.86), COD_RE_ (0.86), and PO_4_^3−^_RE_ (0.91), which reveals that the oxidation of both carbonaceous substrates and NH_3_-N, accumulation of phosphates by PAOs, and accumulation of NO_3_–N due to nitrification were significantly enhanced when more oxic zones were provided by increasing T_a_/T_total_.

The ratios of anoxic periods (T_f_, T_s_, and T_f_ + T_s_) to total batch runtime (T_total_) were evaluated regarding their effects on the SBR performance. Both T_f_/T_total_ and (T_f_ + T_s_)/T_total_ ratios indicated moderate-to-very strong and negative correlations with BOD_5RE_, PO_4_^3−^_RE_, COD_RE_, NH_3_-N_RE_, and NO_3_-N_AR_, revealing that increasing the T_f_ and T_f_ + T_s_ periods weakened the removal efficiencies of BOD_5_, PO_4_^3−^, COD, and NH_3_–N, while the effluent NO_3_-N concentration was reduced by increasing the anoxic periods. On the other hand, the T_s_/T_total_ ratio exhibited moderate and positive correlations with COD_RE_ (0.50), TN_RE_ (0.51), and NO_3_-N_AR_ (0.58). As a general trend, the COD and NO_3_-N removal efficiencies increase, with an increase in T_s_ representing the period of the anoxic zone [24,45,46]. During this anoxic period, the denitrification process is achieved by using the COD as a carbon source and electron donor, and this process is responsible for COD and NO_3_-N removal. In addition, the particulate non-biodegradable fraction of COD can settle down during the sedimentation phase, which can also increase the COD removal rate as T_s_ increases. The reason why a positive correlation between T_f_/T_total_ and COD removal was not obtained could be attributed to the very low influent NO_2_-N and NO_3_-N concentrations, where the effect of the denitrification process on COD consumption is not statistically sound.

### 3.5. Comparison of the SBR System Performance with the Literature

The removal efficiencies of COD, TN, and PO_4_^3−^ achieved by the pilot-scale SBR system with the post-anoxic denitrification scenario in this study were compared to similar reported works in the literature for simultaneous COD and nutrient removal using lab/bench-scale SBR systems. As demonstrated in Table 4, the COD, TN, and PO_4_^3−^ removal efficiencies attained in this research are in good agreement with the literature data. Noticeably, the studies that investigated SBR systems for treating synthetic wastewater influent performed better in terms of the COD, TN, and PO_4_^3−^ removal efficiencies. However, the pilot-scale SBR system with post-anoxic denitrification demonstrated an excellent performance in the removal of COD (91%), nitrogen (83%), and phosphate (90%), in comparison with the others reported for domestic wastewater influent.

The average effluent concentrations of the wastewater quality parameters calculated for NSTP and the obtained pilot-scale SBR system with post-anoxic denitrification in this study were compared with the maximum allowable discharge levels for the treated sewage wastewater imposed by the KSA Ministry of Environment, Water, and Agriculture regulations [48]. It is evident from Table 5 that the effluent concentrations of both NSTP and the proposed SBR system complied with the local standards. The removal efficiencies of turbidity, TSS, TS, and NH_3_–N computed for NSTP and the proposed SBR systems were comparable. However, the proposed SBR system presented herein improved the treatment efficiencies of BOD_5_, COD, TN, and PO_4_^3−^ by 6%, 16%, 17%, and 41%, respectively, in comparison with NSTP. Therefore, the SBR system with a post-anoxic denitrification configuration can be considered as an efficient method for domestic wastewater treatment in terms of the treatment performance, operational simplicity, flexibility in the operational parameters, and cost-effectiveness, by providing anoxic and oxic conditions in the same tank.

## 4. Conclusions

In this work, the establishment and testing of a pilot-scale SBR system were successfully practiced in order to investigate the system performance for the treatment of municipal wastewater samples obtained from the North Sewage Treatment Plant (NSTP) in the Dhahran area of the Eastern Province, KSA. Two scenarios were performed, consisting of pre-anoxic denitrification and post-anoxic denitrification, to study the treatment of wastewater quality parameters. During four months of system operation, satisfactory and stable removal efficiencies of the targeted parameters were achieved. The correlation statistics results revealed that treatment efficiencies of NH_3_-N, TN, PO_4_^3−^, COD, and BOD_5_ were significantly improved by increasing the aeration time fraction in the total SBR runtime, while increasing the total anoxic period in the total SBR runtime improved the NO_3_-N removal efficiency. The treatment efficiencies of turbidity, TSS, and TS indicated very strong and positive correlations with the ratio of the settling period to total batch runtime. The post-anoxic denitrification scenario resulted in the maximum treatment efficiencies of NH_3_-N (99.9%), TN (92.0%), PO_4_^3−^ (90.0%), COD (99.1%), BOD_5_ (89.3%), turbidity (97.9%), TSS (98.8%), and TS (98.4%) when the filling, aeration, sedimentation, and decanting times were set to 0.58, 6.0, 6.0, and 0.20 h, respectively. The effluent concentrations of the targeted wastewater quality parameters computed for the proposed SBR system were below the local standards, and the performance of the proposed SBR system was better than that of NSTP in terms of the BOD_5_, COD, TN, and PO_4_^3−^ treatment efficiencies. Hence, the tested and proposed SBR process is a simple, efficient, flexible, cost-effective, and successful technology for the treatment of municipal wastewaters. The SBR process can be employed in remote areas in arid regions of KSA for wastewater treatment and reuse for sustainable water management.

## Figures and Tables

**Table 1:** Statistical summary of the parameters measured for the influent of the North Sewage Treatment Plant (NSTP).

Parameter *	Unit	Influent				
		Min	Max	Mean	SD	Median
pH	pH unit	7.13	8.06	7.44	0.239	7.35
Turbidity	NTU	34.6	187	81.3	37.8	72.2
TSS	mg L^−1^	283	1,737	887	375	792
TS	mg L^−1^	3187	4482	3810	365	3684
NO_2_–N	mg L^−1^	0.006	0.036	0.015	0.007	0.017
NO_3_–N	mg L^−1^	0.032	0.454	0.165	0.135	0.155
NH_3_–N	mg L^−1^	5.47	27.2	14.6	4.83	14.2
TN	mg L^−1^	9.36	20.8	17.2	3.91	16.9
PO_4_^3−^	mg L^−1^	0.295	4.54	2.23	1.26	2.24
COD	mg L^−1^	68.0	359	180	70.9	179
BOD_5_	mg L^−1^	48.0	144	79.9	25.8	72.0

* Total suspended solids (TSS), total solids (TS), nitrite-nitrogen (NO_2_^–^N), nitrate-nitrogen (NO_3_^–^N), ammonia-nitrogen (NH_3_–N), total nitrogen (TN), phosphate (PO_4_^3−^), chemical oxygen demand (COD), and 5-day biological oxygen demand (BOD_5_).

**Table 2:** Operational conditions of the pilot-scale SBR experiments.

Scenarios *	Batch No *	T_f_ (h)	T_a_ (h)	T_s_ (h)	T_d_ (h)	T_total_ (h)
Pre-denitrification (SCI)	B1-1	8.0	4.0	2.8	0.25	15.1
	B1-2	10.0	4.0	4.0	0.25	18.3
	B2-1	8.0	6.0	4.5	0.33	18.8
	B2-2	12.0	6.0	8.0	0.25	26.3
	B3-1	12.0	4.0	5.0	0.30	21.3
	B3-2	10.0	4.0	4.0	0.30	18.3
Post-denitrification (SCII)	B4-1	0.8	4.0	3.0	0.30	8.1
	B4-2	0.8	3.0	4.0	0.30	8.1
	B5-1	1.0	4.0	8.0	0.25	13.3
	B6-1	0.9	4.0	9.5	0.42	14.8
	B6-2	0.5	4.0	11.0	0.25	15.8
	B6-3	0.6	4.0	6.0	0.28	10.9
	B6-4	0.6	6.0	6.0	0.20	12.8

* SCI: Scenario I, SCII, Scenario II, B1-B6: batch numbers, filling time (T_f_), aeration time (T_a_), and settling time (T_s_).

**Table 3:** Correlation matrix for the removal efficiencies of wastewater quality parameters and operational parameters of the SBR experiments.

^*^Parameters	NH_3_-N_RE_	NO_3_-N_AR_	TN_RE_	COD_RE_	BOD_RE_	PO_4_^3-^_RE_	Turb._RE_	TSS_RE_	TS_RE_
T_f_/T_total_	−0.76	*−0.77*	-	*−0.76*	*−0.56*	*−0.75*	-	-	-
T_a_/T_total_	**0.86**	0.74	0.61	**0.86**	0.51	0.91	-	-	-
T_s_/T_total_	-	0.58	0.51	0.50	-	-	**0.84**	**0.80**	**0.83**
(T_f_+T_s_)/T_total_	***−0.87***	*−0.74*	-	***−0.86***	*−0.51*	***−0.92***	-	-	-

Only *p* ≥ 0.5 and *p* ≤ −0.5 are shown. Negative correlations are shown in italic. Very strong correlations, *p* ≥ 0.8 and *p* ≤ −0.8, are shown in bold, filling time (T_f_), aeration time (T_a_), and settling time (T_s_).

**Table 4:** Comparison of COD, TN, and phosphorus removal efficiencies with the literature.

Wastewater Type	Reactor Volume (L)	* Removal Efficiency (%)			Reference
		COD	TN	Phosphate	
Domestic wastewater	3.5	87	83	74	[36]
Domestic wastewater	5	90	78	56	[32]
Domestic wastewater	10	85	86	82	[41]
Synthetic wastewater	10	97	98	80	[47]
Synthetic sanitary sewer	14	94	96	90	[34]
Domestic wastewater	25	95	78	87	[39]
Domestic wastewater	240	91	83	83	This work

* Chemical oxygen demand (COD), Total nitrogen (TN).

**Table 5:** Comparison of NSTP and SBR effluents and their performances.

* Parameter	Effluent			Discharge Limit (KSA)	Removal Efficiency (%)	
	Unit	NSTP	SBR		NSTP	SBR
pH	pH unit	7.61 ± 0.25	7.87 ± 0.15	[6.0–8.4]	-	-
Turbidity	NTU	2.63 ± 1.31	2.10 ± 0.55	5	97	97
TSS	mg L^−1^	25.2 ± 8.44	13.7 ± 3.51	40	97	98
TS	mg L^−1^	98.8 ± 10.0	78.8 ± 24.9	-	97	98
NO_3_–N	mg L^−1^	3.29 ± 1.20	2.85 ± 2.67	10	-	-
NH_3_–N	mg L^−1^	1.78 ± 2.27	0.98 ± 0.86	5	91	93
TN	mg L^−1^	5.90 ± 1.06	3.13 ± 0.33	-	66	83
PO_4_^3−^	mg L^−1^	1.29 ± 1.13	0.45 ± 0.26	10	42	83
COD	mg L^−1^	44.8 ± 21.6	27.7 ± 16.2	50	75	91
BOD_5_	mg L^−1^	15.0 ± 9.02	11.7 ± 1.97	40	81	87

* Total suspended solids (TSS), total solids (TS), nitrite-nitrogen (NO_2_^–^N), nitrate-nitrogen (NO_3_^–^N), ammonia-nitrogen (NH_3_–N), total nitrogen (TN), phosphate (PO_4_^3−^), chemical oxygen demand (COD), and 5-day biological oxygen demand (BOD_5_), NSTP: North Sewage Treatment Plant, SBR: Seuential bach reactor.
